# Supplementary material for: Targeting vascular dementia: Molecular docking and dynamics of natural ligands against neuroprotective proteins
Source: PLoS One. 2025 Oct 1;20(10):e0331787. doi: 10.1371/journal.pone.0331787 (PMC12488027; doi:10.1371/journal.pone.0331787)
Supplement: S1 File — (DOCX) [file pone.0331787.s001.docx]

# Supplementary Table 1. Docking Score Variability Analysis

Each ligand–protein complex was subjected to five independent docking runs using AutoDock Vina. The table below summarizes the mean binding energies and the standard deviations, indicating the consistency and reliability of docking results.

| **Protein-Ligand** | **Mean Binding Score (kcal/mol)** | **Standard Deviation (kcal/mol)** |
| --- | --- | --- |
| APLP1-Galangin | -6.8 | 1.07 |
| Claudin5-Licocumarone | -5.7 | 0.66 |
| MMP9-Curcumin | -6.66 | 0.99 |
| MTHFR-Resveratrol | -6.32 | 1.03 |
| SOD1-Licocumarone | -6.02 | 0.42 |
| APOE-Curcumin | -6.32 | 0.74 |

# Supplementary Table 2:

# Docking Summary for APLP1 with Galangin

| **Pocket ID** | **Binding Score (kcal/mol)** |
| --- | --- |
| 1 | –8.5 |
| 2 | –6.0 |
| 3 | –6.3 |
| 4 | –6.0 |
| 5 | –7.2 |

# Docking Summary for Claudin-5 with Licocumarone

| **Pocket ID** | **Binding Score (kcal/mol)** |
| --- | --- |
| 1 | –6.8 |
| 2 | –5.5 |
| 3 | –5.6 |
| 4 | –5.0 |
| 5 | –5.6 |

# Docking Summary for Matrix metalloproteinase-9 with Curcumin

| **Pocket ID** | **Binding Score (kcal/mol)** |
| --- | --- |
| 1 | –7.3 |
| 2 | –5.9 |
| 3 | –5.6 |
| 4 | –8.0 |
| 5 | –6.5 |

# Docking Summary for MTHFR with Resveratrol

| **Pocket ID** | **Binding Score (kcal/mol)** |
| --- | --- |
| 1 | –8.1 |
| 2 | –5.7 |
| 3 | –5.9 |
| 4 | –6.3 |
| 5 | –5.6 |

# Docking Summary for Superoxide Dismutase with Licocumarone

| **Pocket ID** | **Binding Score (kcal/mol)** |
| --- | --- |
| 1 | –6.6 |
| 2 | –5.7 |
| 3 | –5.9 |
| 4 | –6.3 |
| 5 | –5.6 |

# Docking Summary for Apolipoprotein E with Curcumin

| **Pocket ID** | **Binding Score (kcal/mol)** |
| --- | --- |
| 1 | –7.2 |
| 2 | –6.6 |
| 3 | –6.7 |
| 4 | –5.5 |
| 5 | –5.6 |
