# Supplementary material for: Targeting vascular dementia: Molecular docking and dynamics of natural ligands against neuroprotective proteins
Source: PLoS One. 2025 Oct 1;20(10):e0331787. doi: 10.1371/journal.pone.0331787 (PMC12488027; doi:10.1371/journal.pone.0331787)
Supplement: Attachment — Submitted filename: Comments for Authors .docx [file pone.0331787.s002.docx]

**Comments for Authors**

This study presents a thorough computational investigation of natural ligands that target proteins associated with vascular dementia (VaD). The analysis incorporates multiple computational approaches, including molecular docking, protein-protein interaction network, and normal mode analysis. The manuscript demonstrates several strengths. Well-organized structure with clear objectives and logical progression. Effective use of various bioinformatics tools (STRING, Enrichr, AutoDock Vina). High-quality graphical abstract and figures that enhance understanding. However, some limitations were noted: Certain sections contain unnecessary redundant explanations, particularly when describing protein functions in VaD. The absence of experimental validation reduces the translational potential of the findings. The discussion section could be improved by better contextualizing the results within the broader field of vascular dementia therapeutics. While the computational approach is sound, the study would benefit from more concise writing and experimental validation to strengthen its conclusions.

**Comments:**

1. Consider a more concise title such as "Targeting Vascular Dementia: Molecular Docking and Dynamics of Natural Ligands Against Neuroprotective Proteins."
2. Streamline the abstract to be more focused and less dense.
3. Explicitly highlight the study's novelty in the abstract, particularly the integration of normal mode analysis with molecular docking.
4. Consolidate descriptions of protein roles (APOE, CLDN5, etc.) that appear redundantly throughout the manuscript.
5. Better articulate the knowledge gap in the introduction, emphasizing the lack of studies combining NMA and docking for VaD therapeutics.
6. Include validation metrics for the DoGSiteScorer active site predictions, such as comparisons with known active sites.
7. Add a comparative table of selected natural compounds (curcumin, galangin, licocumarone, resveratrol) showing relevant properties like bioavailability and previous evidence in neurodegeneration.
8. Explain the relevance of cross-species conservation in the co-expression analysis to human vascular dementia.
9. Provide a biological interpretation of the edge confidence scores in the PPI analysis to clarify their functional significance.
10. Avoid repeating docking scores in the text when they're already presented in Table 3. Instead, summarize key findings more concisely.
11. Include a discussion of structural factors influencing binding affinity between specific ligands and proteins.
12. Link computational findings to potential clinical applications more explicitly in the discussion section.
13. Acknowledge limitations regarding experimental validation and outline future research directions.
14. Focus the discussion on interpretation and implications rather than restating results.
15. Update older references with recent reviews on vascular dementia mechanisms (2022-2024).
16. Ensure consistent formatting of journal names throughout the references.

Major revision required to address redundancy, clarify novelty, and improve clinical relevance. The study has strong potential but needs tighter writing and contextualization. Experimental validation in future work would significantly strengthen the conclusions.
