# Supplementary material for: Targeting vascular dementia: Molecular docking and dynamics of natural ligands against neuroprotective proteins
Source: PLoS One. 2025 Oct 1;20(10):e0331787. doi: 10.1371/journal.pone.0331787 (PMC12488027; doi:10.1371/journal.pone.0331787)
Supplement: Attachment — Submitted filename: Response to Reviewers.docx [file pone.0331787.s003.docx]

Dear Editor and Reviewers,

We would like to sincerely thank you for the time and effort you dedicated to reviewing our manuscript entitled “**Targeting Vascular Dementia: Molecular Docking and Dynamics of Natural Ligands Against Neuroprotective Proteins”.**

We appreciate the insightful and constructive comments provided by the reviewers, which have helped us improve the clarity, rigor, and overall quality of our manuscript. We have carefully considered all suggestions and have addressed each point in detail in the responses below. Changes made in the revised manuscript have been highlighted in yellow.

Below, we provide a point-by-point response to each comment raised by the reviewers, with our detailed explanations and references to the revised manuscript.

**Reviewer #1:** 
Comments:
**1. Consider a more concise title such as "Targeting Vascular Dementia: Molecular Docking and Dynamics of Natural Ligands Against Neuroprotective Proteins."**

**Response:** Thank you for the suggestion. I have made the update based on your suggestion.

**2. Streamline the abstract to be more focused and less dense.**
**Response:** We thank the reviewer for their insightful comment, which helped to enhance the clarity of the study.

**3. Explicitly highlight the study's novelty in the abstract, particularly the integration of normal mode analysis with molecular docking.**

**Response:** We thank the reviewer for their insightful comment. The comment has been addressed appropriately.

**4. Consolidate descriptions of protein roles (APOE, CLDN5, etc.) that appear redundantly throughout the manuscript.**

**Response:** Thank you for bringing this to my attention. The comment has been addressed in the revised manuscript.

**5. Better articulate the knowledge gap in the introduction, emphasizing the lack of studies combining NMA and docking for VaD therapeutics.**

**Response**: Thank you for your thoughtful observation. We have now addressed it in the revised manuscript from lines 96-107.

**6. Include validation metrics for the DoGSiteScorer active site predictions, such as comparisons with known active sites.**

**Response:** We sincerely thank the reviewer for pointing this out and have added the requested details. To validate the active sites predicted by DoGSiteScorer, we cross-referenced them with experimentally resolved structures from the Protein Data Bank (PDB) and corroborated them with published literature. The predicted binding region on APOE aligns with its receptor-binding domain (residues 136–150), confirmed in PDB ID: 1B68 and reported to mediate lipoprotein interactions critical to neurovascular function (1). For MMP9, the predicted pocket includes the catalytic zinc-binding motif (His401, His405, His411), consistent with the structure in PDB ID: 1L6J and literature identifying these residues as essential for proteolytic activity (2). The APLP1 binding region overlaps with the E2 domain observed in PDB ID: 3QMK, previously shown to mediate heparin and amyloid interactions (3). For CLDN5, although no full crystal structure is available, the predicted site spans extracellular loops (ECL1/ECL2), implicated in tight junction formation and blood-brain barrier function (4). Similarly, SOD1’s active site channel, predicted here and visualized in PDB ID: 5YTU, includes conserved histidines coordinating Cu/Zn ions, corroborated by (5). Lastly, MTHFR’s ligand pocket overlaps with the FAD-binding catalytic domain (PDB ID: 6FCX), as functionally characterized in oxidative stress and homocysteine regulation (6). These validations affirm the biological accuracy of the predicted docking sites and strengthen the reliability of the subsequent molecular docking results.

1.	Chen F, Zhao J, Meng F, He F, Ni J, Fu Y. The vascular contribution of apolipoprotein E to Alzheimer’s disease. Brain. 2024;147(9):2946-65.

2.	Azargoonjahromi A, Eivazi M, Nasiri H, Tarhriz V, Payandeh Z, Initiative AsDN. Elevated CSF GAP-43 in Mild Cognitive Impairment Linked to Cognitive Impairment Through Increased Amyloid-β Accumulation, with a Shift to Reduced Amyloid-β Accumulation in Alzheimer’s Disease. Journal of Molecular Neuroscience. 2025;75(2):39.

3.	Onodera W, Asahi T, Sawamura N. Rapid evolution of mammalian APLP1 as a synaptic adhesion molecule. Scientific reports. 2021;11(1):11305.

4.	Hashimoto Y, Poirier K, Boddaert N, Hubert L, Aubart M, Kaminska A, et al. Recurrent de novo mutations in CLDN5 induce an anion-selective blood–brain barrier and alternating hemiplegia. Brain. 2022;145(10):3374-82.

5.	Tiwari MK, Hägglund PM, Møller IM, Davies MJ, Bjerrum MJ. Copper ion/H2O2 oxidation of Cu/Zn-Superoxide dismutase: Implications for enzymatic activity and antioxidant action. Redox biology. 2019;26:101262.

6.	Sree GNS H, Chandramohan V, Rajalekshmi SG, Burri RR. Computational investigations of structural and interactive patterns of human MTHFR wild and mutant type under different physiological conditions: An insight into underlying perturbations. Alzheimer's & Dementia. 2021;17:e053328.

**7. Add a comparative table of selected natural compounds (curcumin, galangin, licocumarone, resveratrol) showing relevant properties like bioavailability and previous evidence in neurodegeneration.**

**Response:** We are grateful for the constructive feedback. In response, Table 3 has been added to the results section.

**8. Explain the relevance of cross-species conservation in the co-expression analysis to human vascular dementia.**

**Response:** We thank the reviewer for this insightful comment. We have now clarified the relevance of cross-species conservation in the co-expression analysis, see revised text in the Discussion section being highlighted. Specifically, we explain that observing conserved co-expression patterns of APOE, APLP1, MMP9, CLDN5, SOD1, and MTHFR across humans and model organisms (e.g., *Mus musculus*, *Danio rerio*) highlights their evolutionary stability and functional significance. These genes are involved in fundamental processes such as vascular regulation and neuroinflammation mechanisms central to the pathogenesis of vascular dementia (VaD). The conservation suggests that the biological roles of these genes are likely preserved in humans, enhancing the translational relevance of our findings and supporting their selection as potential therapeutic targets. We have incorporated this explanation to strengthen the rationale behind our gene selection.
**9. Provide a biological interpretation of the edge confidence scores in the PPI analysis to clarify their functional significance.**

**Response:** We thank the reviewer for this valuable comment. To clarify the biological significance of the edge confidence scores in the PPI analysis, we have added a paragraph explaining how these scores reflect the reliability and functional strength of protein–protein interactions and are highlighted. We also interpreted high-confidence interactions (score > 0.7) as indicators of well-supported regulatory links relevant to vascular dementia pathogenesis.

**10. Avoid repeating docking scores in the text when they're already presented in Table 3. Instead, summarize key findings more concisely.**

**Response:** We appreciate the reviewer’s valuable suggestion and have revised the section accordingly.

**11. Include a discussion of structural factors influencing binding affinity between specific ligands and proteins.**

**Response:** We appreciate the reviewer’s comment and have now included a detailed discussion in the revised manuscript in discussion page 34, explaining how structural features such as ligand planarity, hydrogen bonding capacity, binding pocket architecture, and presence of metal ions influence binding affinity. These interpretations are based on our docking and LigPlot interaction data and provide molecular-level rationale for the observed differences in docking scores.

**12. Link computational findings to potential clinical applications more explicitly in the discussion section.**

**Response:** We thank the reviewer for this important suggestion. In response, we have added a paragraph to the Discussion section on page 35 explicitly linking our computational findings to potential clinical applications. We now highlight how the identified ligands and pathways relate to known therapeutic targets and biological mechanisms in vascular dementia, and how these natural compounds could be advanced as multi-target agents for future preclinical or clinical investigation.

**13. Acknowledge limitations regarding experimental validation and outline future research directions.**

**Response:** We thank the reviewer for their insightful comment, and it has been addressed in the manuscript form lines 617-619.

**14. Focus the discussion on interpretation and implications rather than restating results.**

**Response:** Thank you for your thoughtful observation. We have now addressed it in the revised manuscript.

**15. Update older references with recent reviews on vascular dementia mechanisms (2022-2024).**

**Response:** We appreciate the reviewer’s valuable suggestion and have revised the section accordingly.

**16. Ensure consistent formatting of journal names throughout the references.**

**Response:** We are grateful for the constructive feedback, which helped us improve the clarity.
