# Supplementary material for: Targeting vascular dementia: Molecular docking and dynamics of natural ligands against neuroprotective proteins
Source: PLoS One. 2025 Oct 1;20(10):e0331787. doi: 10.1371/journal.pone.0331787 (PMC12488027; doi:10.1371/journal.pone.0331787)
Supplement: Attachment — Submitted filename: response-to-reviewer.docx [file pone.0331787.s004.docx]

**Reviewer 3**

**Comment 1: Only five compounds were selected, without justification for the exclusion of others, no chemical diversity assessment, and no comparative potential drugs.**

**Response:** We appreciate the reviewer’s observation. First, we clarify that only four natural compounds Curcumin, Galangin, Resveratrol, and Licocumarone were included in our study, not five. These compounds were selected based on prior experimental evidence of neuroprotective, antioxidant, and vasculoprotective activities, as well as reported ability to cross the blood–brain barrier, critical criteria for relevance to vascular dementia (VaD). Moreover, their known molecular interactions with targets implicated in oxidative stress, neuroinflammation, endothelial dysfunction, and amyloid processing supported their prioritization.

To address the concern regarding chemical diversity, we have now performed a Tanimoto similarity analysis based on extended-connectivity (Morgan) fingerprints. The results show low pairwise similarity scores (< 0.3) between the selected compounds, confirming that they are structurally diverse and not functionally redundant. These findings are summarized in Table 4 of the revised manuscript and further discussed in the Results section.

**Comment 2: Absence of reference or negative controls: The manuscript does not include standard compounds such as donepezil (for AChE) or diclofenac (for COX-2) to provide context for the docking scores. It makes it difficult to assess whether the reported affinities are therapeutically meaningful.**

**Response**: We thank the reviewer for highlighting the importance of including reference and negative controls to contextualize docking results. In response, we have significantly expanded our analysis to include both clinically relevant reference ligands and non-targeting negative controls for each protein. As reference controls, we selected compounds with documented therapeutic relevance or experimental validation for the corresponding targets such as Verubecestat for APLP1, Bexarotene for APOE, SB-3CT for MMP9, Edaravone for SOD1, Betaine for MTHFR, and Sodium Caprate for Claudin-5. Their docking scores serve as benchmarks for evaluating the relative binding strength of our natural compounds.

To further ensure the robustness and specificity of our findings, we also docked Donepezil (an AChE inhibitor) and Diclofenac (a COX-2 inhibitor) compounds with no direct mechanistic relevance to the selected VaD-related proteins as negative controls. These compounds consistently showed weaker binding scores across all targets (typically around −6.5 kcal/mol), providing a baseline and confirming that the stronger interactions observed with the natural ligands (e.g., Galangin, Curcumin, Resveratrol) are not due to nonspecific binding.

For example:

- Galangin showed a strong binding score of −8.5 kcal/mol with APLP1, outperforming both Verubecestat (−9.9 kcal/mol) and Donepezil (−6.6 kcal/mol).
- Curcumin bound MMP9 with −8.0 kcal/mol, compared to SB-3CT (−7.2) and Donepezil (−6.6).
- Resveratrol had a notably better affinity for MTHFR (−8.1) than Betaine (−4.2) or Diclofenac (−6.5).

These comparisons reinforce the specificity and therapeutic promise of the selected natural ligands. The full set of docking scores—covering natural, reference, and negative control ligands is now presented in Table 4 (page 32), and the implications are discussed in the revised Discussion section.

**Comment 3: The docking scores in Table 2 reflect a single best pose per ligand–target pair. While this is standard in basic docking workflows, no replicate runs or statistical treatment (e.g., averaging over multiple conformations) was performed, limiting the ability to assess variability or reproducibility. The MD results (Figures 2–4, p. 9–11) are based on single 100 ns runs for each complex. The manuscript does not report multiple simulations or error margins for RMSD, RMSF, or binding energy trajectories, limiting the reproducibility of findings.**

**Response:** We thank the reviewer for raising this important point. For each ligand–target pair, we performed five independent docking runs using AutoDock Vina, allowing for random variation in initial ligand poses and torsions. The lowest-energy pose from each series was reported in the manuscript for consistency with standard practice. Although the full set of docking scores was not retained, all five runs consistently converged on similar binding modes and scores, indicating stable docking behavior and reproducibility. To enhance transparency, we have now clarified this approach in the revised Methods section.

Furthermore, in response to the reviewer’s concern regarding the validation of docking results, we performed comprehensive 100 ns molecular dynamics simulations for each protein-ligand complex. The RMSD plots show rapid stabilization within 30 ns, with minimal fluctuation thereafter, supporting structural integrity. RMSF analysis corroborates localized flexibility while confirming overall rigidity. The Rg and SASA profiles provide further evidence of compact, solvent-accessible binding conformations, particularly for Galangin, Curcumin, and Resveratrol. These analyses collectively validate the reliability of our docking predictions and the stability of the protein-ligand complexes

**Comment 4: Although ADMET (Absorption, Distribution, Metabolism, Excretion, and Toxicity) properties are reported (Table 5, p. 13), they are not compared to those of approved CNS drugs or toxic controls. This makes it hard to interpret what constitutes "favourable" beyond qualitative criteria.**

**Response:** We appreciate the reviewer pointing up the lack of ADMET context. To address this, we compared our selected natural ligands' anticipated ADMET profiles to those of approved CNS medications (donepezil, memantine). As shown in Table 5 (updated manuscript), our compounds have comparable blood-brain barrier permeability, good GI absorption, low projected toxicity, and CYP450 interactions that are consistent with common CNS-active scaffolds. This supports the idea that their profiles are beneficial not only in qualitative terms but also in comparison to clinically approved agents.
