# Supplementary material for: Targeting vascular dementia: Molecular docking and dynamics of natural ligands against neuroprotective proteins
Source: PLoS One. 2025 Oct 1;20(10):e0331787. doi: 10.1371/journal.pone.0331787 (PMC12488027; doi:10.1371/journal.pone.0331787)
Supplement: Attachment — Submitted filename: response_to_reviewers_auresp_3.docx [file pone.0331787.s005.docx]

**Comment 1: The "Materials and Methods" section states that each ligand was docked independently five times, and the pose with the lowest binding energy was chosen. To enhance the reproducibility and confidence in the docking results, the manuscript should explicitly include details on the variability across these five runs in the "Results" section, such as the standard deviation of the binding energies or a statement about the consistency of the top poses. This would provide greater insight into the stability of the predicted binding modes.**

**Response:** We appreciate the reviewer’s insightful suggestion. We have now clarified in the Materials and Methods section that each protein–ligand complex was docked five times using AutoDock Vina.

In response to the comment, we performed a statistical analysis of the docking scores, and the standard deviations for each set of five runs have been calculated and are presented in Supplementary Table 1. Additionally, the Results section now includes a detailed summary of these values, demonstrating the consistency and low variability of the docking results (SD range: 0.42–1.07 kcal/mol). This addition strengthens the reproducibility and confidence in our docking protocol.

**Comment 2: The study performed 100 ns molecular dynamics simulations for each protein-ligand complex, analyzing RMSD, RMSF, Rg, and SASA. While 100 ns is a reasonable duration, the absence of multiple independent simulation runs or statistical reporting (e.g., error margins) for these metrics within the main text limits the assessment of the reproducibility of the dynamic behavior. Including such data would significantly strengthen the claims of structural stability and conformational behavior.**

**Response:** We appreciate the reviewer’s insightful suggestion regarding statistical robustness in molecular dynamics (MD) simulations. While we conducted one 100 ns MD simulation per complex due to computational constraints, we have now revised the manuscript to include post-equilibration (50–100 ns) averaging and variation analysis for RMSD, Rg, and SASA to assess structural consistency. The revised section (in Results) emphasize that low standard deviations (<0.06 nm RMSD; ±0.04 nm Rg) across the final 50 ns confirm structural convergence and minimize stochastic variation. This addition supports the stability of the observed conformational behavior despite the absence of replicates.

**Comment 3: Table 5 summarizes the ADMET properties of the selected natural compounds. While beneficial, the manuscript does not adequately contextualize these properties against clinically approved CNS drugs within the main "Results" or "Discussion" sections. Explicitly discussing how the "favorable CNS drug-likeness and safety profiles" compare to existing medications in the main text would provide a clearer benchmark for their therapeutic potential.**

**Response:** We agree with the reviewer’s recommendation to provide a clearer contextualization of the ADMET results. The Results section now includes a more detailed comparison of the natural ligands to Donepezil and Memantine, addressing their blood–brain barrier permeability, GI absorption, and safety margins. We also expanded the Discussion to reflect how these compounds align with established pharmacokinetic criteria for CNS drug development. These clarifications now highlight the therapeutic potential and translational relevance of the selected natural molecules.	**Comment 4: The manuscript does not present comparative docking data with reference (positive) and negative controls for the protein targets within the main "Results" section. This information is critical for validating the specificity and therapeutic relevance of the natural ligands by allowing readers to immediately assess the strength of the natural ligands' binding affinities against established benchmarks. For example, comparing the binding of natural ligands to known inhibitors or non-binding compounds would significantly strengthen the interpretation of the docking scores.**

**Response:** Thank you for this insightful recommendation. We agree that comparative analysis is critical for validating the docking results. While Table 7 presented docking scores with reference inhibitors and non-specific compounds, we have now expanded the main Results section to include a detailed comparative discussion. This addition highlights that natural ligands like Curcumin, Galangin, and Licocumarone often show stronger or comparable binding than clinically known inhibitors, such as SB-3CT, Edaravone, or Verubecestat, while outperforming negative controls like Donepezil and Diclofenac. This reinforces the specificity, potency, and therapeutic relevance of our natural compounds.
**Comment 4: The statistical analysis appears mostly appropriate for the reported data. Pathway enrichment analyses correctly utilize p-values and q-values for statistical significance and false discovery rate correction, respectively. However, the rigor could be enhanced by explicitly addressing the statistical treatment of the docking scores (e.g., reporting mean and standard deviation from the five runs) and the molecular dynamics simulation metrics. Without this, the statistical robustness of these specific sections remains somewhat open to interpretation.
Response:** We appreciate the reviewer’s emphasis on enhancing statistical transparency. In response, we have now included mean binding energies and standard deviations for all docking simulations, derived from five independent AutoDock Vina runs per protein–ligand complex. This data is presented in the Results section and detailed in Supplementary Table 1. Additionally, we improved the treatment of molecular dynamics data by applying time-windowed averaging (last 50 ns) and reporting the observed variability for metrics such as RMSD, radius of gyration, and SASA. These changes ensure that both docking and simulation analyses are presented with greater statistical rigor and reproducibility.

**Addional Comments**

**Comment 1: While Table 1 outlines the roles of proteins in VaD, a more detailed explanation of how the KEGG pathway analysis specifically led to the selection of these six proteins among others involved in VaD would be beneficial. What specific criteria from the KEGG analysis made them the optimal targets?**

**Response:** Thank you for this helpful suggestion. We have now clarified the rationale for protein selection in the Methods sections. The six proteins were chosen based on a multi-step filtering process combining:

- KEGG pathway enrichment overlap,
- High PPI network centrality,
- VaD-specific functional roles from literature, and
- Co-expression conservation across species.

This ensures that the selected targets are not only statistically enriched but also biologically central to vascular dementia pathophysiology, thus justifying their prioritization for structural modeling and docking.

**Comment 2: The discussion mentions that findings warrant further experimental validation. Providing specific examples of in vitro (e.g., binding assays, enzyme activity assays) or in vivo (e.g., animal models of VaD to assess cognitive improvements, BBB integrity) experiments would provide a clearer roadmap for future research and enhance the translational relevance of the study.**

**Response:** Thank you for this insightful suggestion. We have expanded the Discussion section to outline specific experimental approaches that could follow this computational study. These include in vitro binding and enzyme inhibition assays, cell-based BBB integrity models, and in vivo testing in animal models of vascular dementia such as chronic cerebral hypoperfusion or BCCAO. These additions provide a clearer translational roadmap and enhance the real-world relevance of our findings.
